# Primary URECs: a source to better understand the pathology of renal tubular epithelia in pediatric hereditary cystic kidney diseases

**DOI:** 10.1186/s13023-022-02265-1

**Published:** 2022-03-09

**Authors:** Wolfgang H. Ziegler, Sarah Lüdiger, Fatima Hassan, Margarita E. Georgiadis, Kathrin Swolana, Amrit Khera, Arne Mertens, Doris Franke, Kai Wohlgemuth, Mareike Dahmer-Heath, Jens König, Claudia Dafinger, Max C. Liebau, Metin Cetiner, Carsten Bergmann, Birga Soetje, Dieter Haffner

**Affiliations:** 1 Department of Pediatric Kidney, Liver and Metabolic Diseases, Hannover Medical School, Hannover, Germany; 2 Department of General Pediatrics, University Children’s Hospital Münster, Münster, Germany; 3 Department of Pediatrics and Center for Molecular Medicine, University Hospital Cologne and Faculty of Medicine, University of Cologne, Cologne, Germany; 4 Center for Rare Diseases, University Hospital Cologne and Faculty of Medicine, University of Cologne, Cologne, Germany; 5 Department of Pediatric Nephrology, Pediatrics II, University of Duisburg-Essen, Essen, Germany; 6 Department of Medicine IV, Faculty of Medicine and Medical Center, University of Freiburg, Freiburg, Germany; 7 Medizinische Genetik Mainz, Mainz, Germany; 8 Present Address: Department of Systemic Cell Biology, Max Planck Institute of Molecular Physiology, Dortmund, Germany

**Keywords:** Urine-derived renal tubular epithelial cells (URECs), Hereditary cystic kidney diseases, Children, 3D culture, Spheroids, Epithelial morphogenesis, Personalized medicine

## Abstract

**Background:**

In pediatric hereditary cystic kidney diseases, epithelial cell defects mostly result from rare, autosomal recessively inherited pathogenic variants in genes encoding proteins of the cilia-centrosome complex. Consequences of individual gene variants on epithelial function are often difficult to predict and can furthermore depend on the patient’s genetic background. Here, we studied urine-derived renal tubular epithelial cells (URECs) from genetically determined, pediatric cohorts of different hereditary cystic kidney diseases, comprising autosomal recessive polycystic kidney disease, nephronophthisis (NPH) and the Bardet Biedl syndrome (BBS). UREC characteristics and behavior in epithelial function-related 3D cell culture were compared in order to identify gene and variant-specific properties and to determine aspects of epithelial (cell) dysfunction.

**Results:**

UREC preparations from patients (19) and healthy controls (39) were studied in a qualitative and quantitative manner using primary cells cultured for up-to 21 days. In patients with biallelic pathogenic variants in *PKHD1* or *NPHP* genes, we were able to receive satisfactory amounts of URECs of reproducible quality. In BBS patients, UREC yield was lower and more dependent on the individual genotype. In contrast, in UREC preparations derived from healthy controls, no predictable and satisfactory outcome could be established. Considering cell proliferation, tubular origin and epithelial properties in 2D/3D culture conditions, we observed distinct and reproducible epithelial properties of URECs. In particular, the cells from patients carrying *PKHD1* variants were characterized by a high incidence of defective morphogenesis of monolayered spheroids—a property proposed to be suitable for corrective intervention. Furthermore, we explored different ways to generate reference cell lines for both—patients and healthy controls—in order to eliminate restrictions in cell number and availability of primary URECs.

**Conclusions:**

Ex vivo 3D cell culture of primary URECs represents a valuable, non-invasive source to evaluate epithelial cell function in kidney diseases and as such helps to elucidate the functional consequences of rare genetic disorders. In combination with genetically defined control cell lines to be generated in the future, the cultivation of primary URECs could become a relevant tool for testing personalized treatment of epithelial dysfunction in patients with hereditary cystic kidney disease.

**Supplementary Information:**

The online version contains supplementary material available at 10.1186/s13023-022-02265-1.

## Background

Hereditary cystic kidney diseases, a heterogenous group of rare disorders affecting both kidneys and eventually other organs, are characterized by pathogenic variants in genes of the cilia-centrosome complex [1–3]. So far, more than 70 causative genes have been identified leading to monogenetic disorders that often manifest in early infancy or childhood. In this age group, the autosomal recessive polycystic kidney disease (ARPKD), nephronophthisis (NPH) and NPH-related ciliopathies (NPH-RC) as well as the Bardet–Biedl syndrome (BBS) are the clinically most relevant disorders as they represent the most frequent genetic causes of end-stage kidney disease (ESKD) in the first 2 decades of life [2, 4, 5]. In contrast, the far more abundant autosomal dominant polycystic kidney disease (ADPKD) is typically characterized by an onset of clinical symptoms in adulthood. So far, no curative or targeted treatment is available for most of these diseases. Consequently, the majority of NPH patients and about half of the ARPKD patients require kidney replacement therapy already before reaching adult age [5, 6].

To understand and to define the functional consequences of the various underlying genetic variants is an important and often rather difficult aspect of hereditary cystic kidney diseases [2, 7]. Apart from a classical loss-of-function owing to a complete gene deletion or non-sense mutations, also missense variants and small deletions may lead to severely impaired gene and protein function. Particularly in large genes such as the polycystic kidney and hepatic disease gene 1 (*PKHD1*; ≥ 479 kb) causing ARPKD, a multitude of private variants has been described [6]. Above that, the genetic origin of other hereditary cystic kidney diseases like NPH and BBS is highly polygenic and the number of identified causative genes is still growing constantly [2, 5]. However, despite this tremendous variety of genetic variants, evoked consequences for epithelial cell function appear to follow similar rules in many of the underlying cystic kidney diseases and might even converge on some common pathways leading to defective epithelial homeostasis and cyst formation [2, 5, 8, 9].

Loss of gene function in hereditary cystic kidney diseases is associated with defective homeostasis of the kidneys’ tubular epithelia, or embryonal development in the most severe cases, and in some of the disease entities affects epithelia of additional organs, for example bile duct epithelia of the liver in ARPKD and NPH. Although molecular mechanisms of disease development are poorly understood and may involve a number of different pathways, cystic expansions, as observed in different segments of the nephron, or loss of epithelia are generally assumed to significantly contribute to progressive decay of kidney function [3, 5, 9]. Pathogenic variants of disease-causing genes are expected to result in cell-autonomous defects of renal epithelial cells, thus providing the rationale for ex vivo investigation of patient-derived cells.

Material to study epithelial function based on biopsies or tissue samples from nephrectomies is for obvious reasons rather limited in pediatric patients, and in times of widely-available comprehensive genetic testing usually not required for diagnostic purposes. Recently, a non-invasive method to collect primary urine-derived renal tubular epithelial cells (URECs) has been developed and studies focused on individual variants suggest that patient-derived URECs can be a suitable source of cells to analyze consequences of disease genetics—such as defective splicing and severely reduced mRNA levels—and to assess defective epithelial cell function [10–13].

Epithelial cell models, often in conjunction with animal models, are widely employed in attempts to define cell biological consequences of ciliopathy gene variants on epithelial function. However, there still is a need for models based on human renal epithelial cells with proven disease outcome, since genetic models in animals frequently show differing disease characteristic and consequences of mutation can vary with individual genotype [2, 7, 14].

To address epithelial cell properties, formation of monolayered, polarized epithelial spheroids (also called cysts or acini) in 3D culture is an accepted model to test cells’ capacity to perform epithelial morphogenesis in culture. This model can also be used to study consequences of pharmacological interventions [15–18]. The use of primary cultures, such as URECs, in cell biological experiments, however, requires control of basic cellular features in every preparation and is limited by survival and proliferation of cells, restricting their use to a total of 3 weeks in culture with 7–10 days for assays.

Here, we addressed the questions, whether URECs from pediatric patients with ARPKD, NPH or BBS can be harvested at sufficient amounts, initially characterized and utilized in biological tests, to define and better understand epithelial function in kidney pathology. Second, we asked whether selected UREC properties reporting epithelial cell dysfunction are associated with certain disease entities and/or individual genetic variants.

## Results

**Fig. 1:**
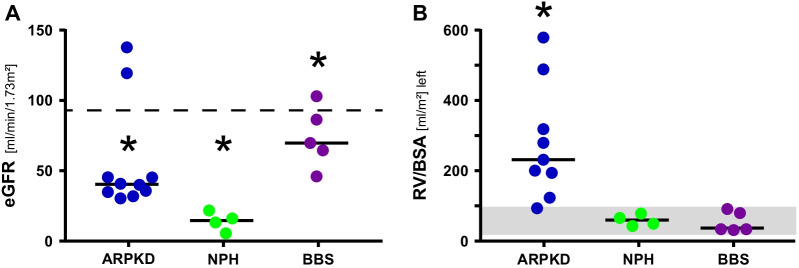
Distribution of kidney parameters within patient cohorts. Filled circles represent values of eGFR (**A**) and left kidney volume (RV/BSA) (**B**) for individual patients as determined by methods adjusted for children [20, 21]. Median values of cohorts, values for normal kidney function (estimated glomerular filtration rate (eGFR) > 90 ml/min/1.73 m^2^) and normal range of body-surface-area related renal volume (RV/BSA, 36–94 ml/m^2^) [21] are indicated. When disregarding two ARPKD patients with normal kidney function as statistical outliers, eGFR values are normally distributed and eGFR significantly differ between cohorts, (*p* < 0.05, ANOVA, Tukey’s). Kidney volumes of ARPKD patients show a wide distribution, with similar size of left and right organs, **(B)** and Additional file 1: Fig. S1, and differ from those determined for NPH and BBS patients (*p* < 0.05, Kruskal–Wallis, Dunn’s). Size of right kidneys and total kidney volumes are provided in Additional file 1: Fig. S1

With support of the German Network for Early Onset Cystic Kidney Disease (NEOCYST), we collected urine samples from genetically confirmed pediatric patients with ARPKD, NPH and BBS [19]. In the study cohort, 16 male and 3 female patients aged 3–17 years were included owing to access to spontaneous urine and successful culture of cells, as well as 39 age-matched healthy controls with even distribution of male and female donors. Clinical characteristics of the study cohort including ultrasound appearance of the kidneys and kidney function are displayed in Table 1 and Fig. 1. For each patient, genetic information and the corresponding estimated glomerular filtration rate (eGFR) are provided in Additional file 1: Table S1.

**Table 1:** Characteristics of patient cohorts and controls providing urine for UREC preparations

Phenotype	Controls (n = 39)	ARPKD (n = 10)	NPH (n = 4)	BBS (n = 5)
Gene(s)	None	*PKHD 1*	*NPHP* (− 1, − 3)	*BBS* (− 1, − 4, − 7, − 10)
Age (years)	8 (1–22)	10 (3–17)	9 (5–13)	14 (9–17)
Sex (male/female)	20/19	7/3	4/0	5/0
eGFR (ml/min/1.73 m^2^)	n.d	40 (30–137)	14 (5–21)	69 (46–103)
Renal volume/body surface area (ml/m^2^)	n.d	225 (89–575)	55 (33–77)	47 (28–90)

Parameters of cohorts with size of groups, n = 4–10 individuals; controls include pooled probes and represent n = 39 individuals. Data sets are given as median (range). Clinical parameters of patients summarize estimated glomerular filtration rate (eGFR) and organ size (RV/BSA) of both kidneys. Statistics are given in legend of Fig. 1. *n.d*, not determined

**Fig. 2:**
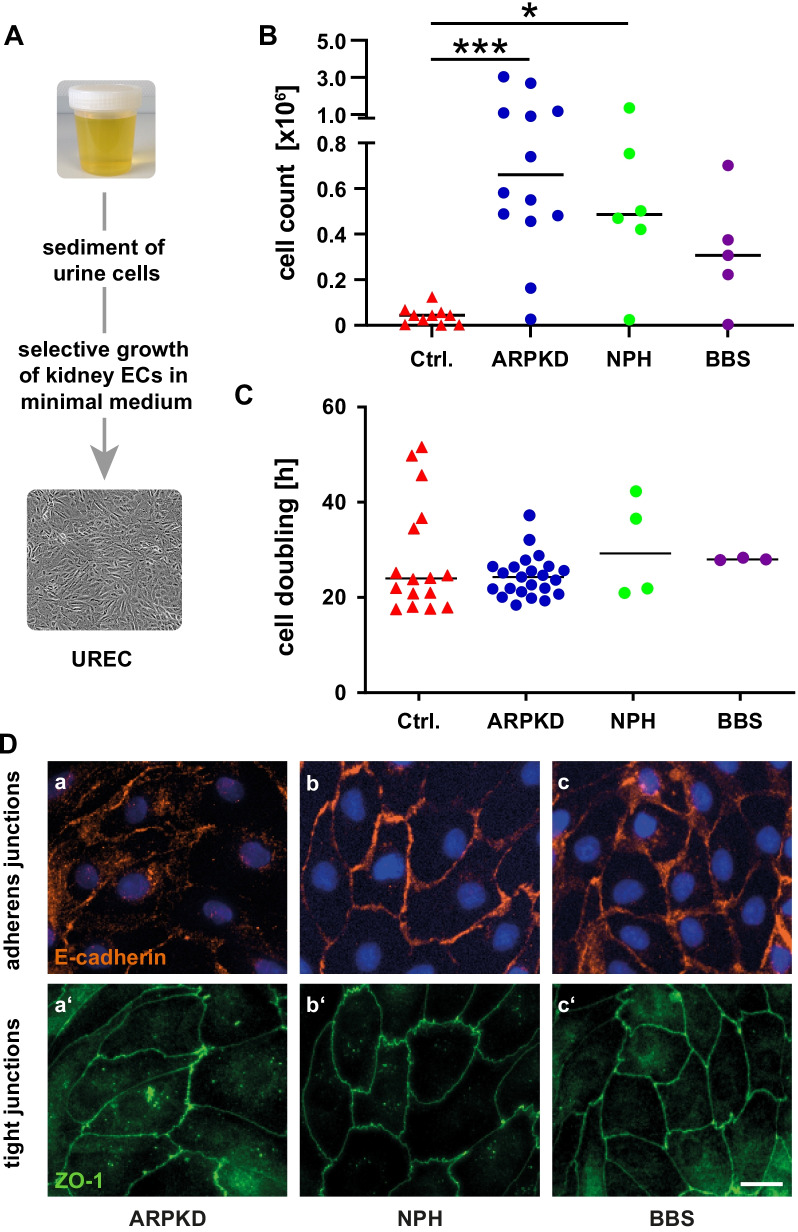
UREC preparations from patients with hereditary cystic kidney diseases and controls, yield and properties in 2D culture. **A** Scheme of UREC culture based on cell harvest from 30 to 50 ml of spontaneous urine and establishment of renal tubular epithelial cell colonies. **B** Yield of independent UREC preparations after 13–15 days in culture, as indicated by filled circles, is highly variable and most efficient in ARPKD and NPH cohorts. Repeat preparations for patients are included. Note low outcome for controls (filled triangles), single children (and pools) with no cystic kidney genetics and normal organ function. Median values of cell count are indicated. (Kruskal–Wallis, Dunn’s; */*** for *p* < 0.05/*p* < 0.001). **C** Population doubling time in 2D culture of spare to medium dense colonies was determined based on metabolic activity of cells growing in medium with 0.5% serum. Median times of cell doubling are indicated and no significant differences observed (Kruskal–Wallis). **D** Representative UREC cultures showing formation of cell–cell junctions in dense 2D culture, as stained for E-cadherin (red, **a**–**c**) and for zonula occludens 1 (ZO-1; green, **a′**–**c′**) in corresponding micrographs. There was individual variability in signal strength but no evidence observed for defects in formation of adherens or tight junctions in any of the preparations. Size bar 20 µm

UREC preparations followed an adapted protocol published by Ajzenberg and Giles [11, 22] using 30–50 ml of participants’ spontaneous urine. In infants and toddlers, urine volumes of 10 ml volumes were also sufficient. Preparation of URECs involved culture of exfoliated cells in a selective minimal medium, allowing establishment and survival of epithelial cell colonies, which were passaged not more than two times before usage in epithelial cell function-related assays, Fig. 2A. To collect samples from different centers, we devised a method allowing preservation and shipment of primary urine cells, up-to 24 h at ambient temperature, before start of cell culture. UREC preparations of the ARPKD and NHP cohorts, including repeated sampling, showed success rates of 70–90%, while in BBS patients successful preparations were only achieved in less than 50%. The median yield of cells after 13–15 days of culture, was 580,000 for the ARPKD cohort and 490,000 cells for NPH. For the BBS cohort a median yield of 310,000 cells was observed, Fig. 2B.

In contrast, in healthy controls relevant amounts of cells could be achieved in no more than 5% of urine samples. Despite > 200 individual preparations, additional increase of urine volume and even pooling of multiple samples, the amount of yielded control cells was significantly lower compared to disease cohorts, ARPKD and NPH, with a median of 44,000 cells only (*p* < 0.001/0.05), Fig. 2B.

To determine and to compare proliferation of URECs, population doubling of cells growing in sparse to medium dense colonies and low serum conditions (0.5% FBS) was monitored for 60 h and calculated based on metabolic activity measurements. This revealed a median duration of cell doubling of 24 h for ARPKD-originated cells, while the duration for NPH and BBS cohorts were 29 and 28 h, respectively. There was no significant difference observed between individual groups, neither between disease cohorts nor in comparison to healthy controls, Fig. 2C.

To address properties of epithelial monolayers in 2D, cell preparations were grown in confluent culture with subsequent immunofluorescent staining of different marker proteins to determine cell origin as well as control of cell–cell junctions and epithelial polarity. UREC monolayers were stained for adherens junctions (E-cadherin, β-catenin), tight junctions (ZO-1) and markers of tubular origin, aquaporin 1 and 2. After 16–18 days in culture, all preparations from patient cohorts were positive for aquaporin 2, reflecting a collecting duct origin of URECs. In addition, some preparations of ARPKD and BBS patients were also positive for aquaporin 1, a marker of the proximal tube, which may suggest a mixture of origin or alternatively loss of differentiation in cell culture conditions (data not shown). Furthermore, regular cell–cell adhesion revealed by adherens junction and tight junction formation was observed in all preparations from all three patient cohorts as displayed by representative micrographs in Fig. 2D.

To determine epithelial properties in 3D culture, a defined number of single cells in suspension was seeded into matrigel (1) to monitor proliferation in close-to in vivo conditions and (2) to study formation of monolayered epithelial spheroids with apicobasal polarity—a measure of cells’ capacity to perform epithelial morphogenesis [15, 16]. 3D structures obtained within 6 days of culture were fluorescently labelled for epithelial markers and captured in stacks of 4-colour images. Stacks were evaluated in a blinded manner by at least 3 independent raters, as described earlier [23]. Analysis of spheroids was based on staining of nuclei (number, 3D position), apical actin belt (force), E-cadherin (cell–cell junction) and acetylated tubulin (apical, cilia).

The proliferative capacity—as required for the assay, which leads to structures of 8–20 cells deriving from a single UREC within the 6-day period—was observed in preparations of only a limited number of patients: 6 participants with ARPKD, 2 with NPH (both carrying a homozygous *NPHP1* deletion) and 3 displaying a Bardet Biedl syndrome (with variants of *BBS4* and *BBS7*). Noteworthy, repetitive analysis of independent UREC preparations from the same participant (5 of 6 ARPKD patients and 1 NPH patient) showed reproducible outcome measures—thus confirming reliability of values determined for respective patients and the cohorts.

**Fig. 3:**
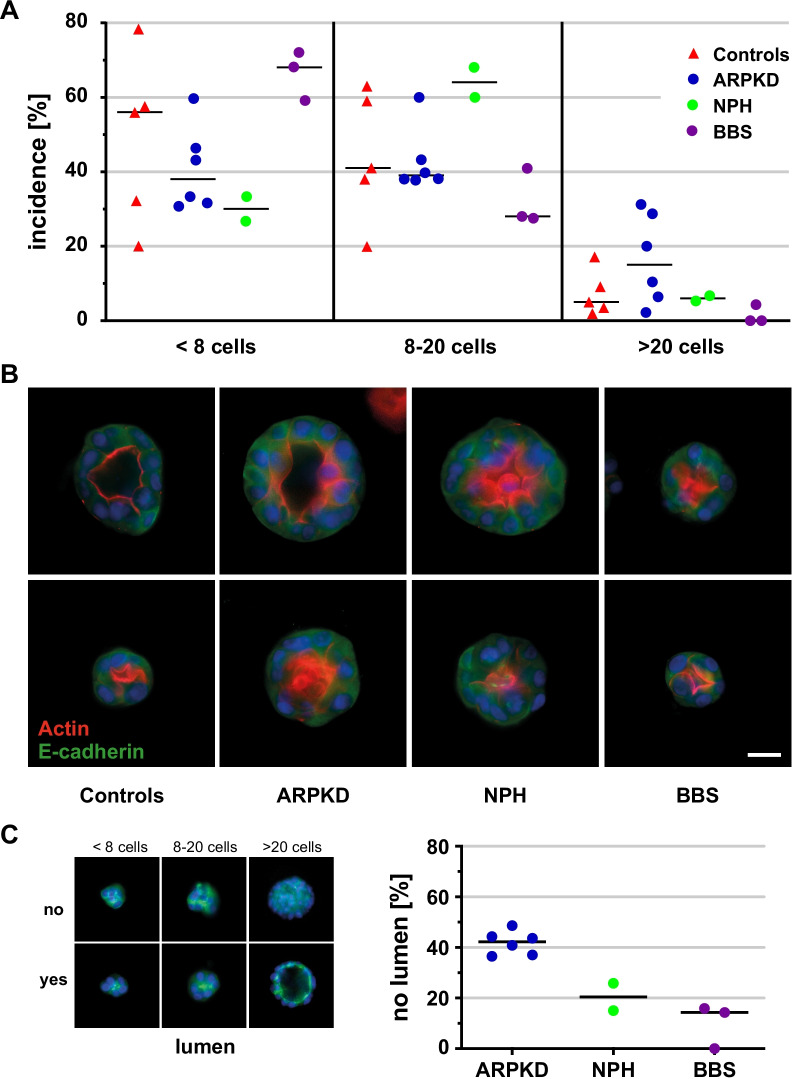
3D culture of URECs from patients with hereditary cystic kidney diseases, proliferation in matrigel and formation of polarized epithelia. Epithelial spheroids in matrigel, originating within 6 days from a single UREC, were rated based on 3D-structure and four fluorescent markers. **A** Size distribution, relative incidence of 3D structures with low number (< 8 cells, left), regular proliferation (8–20 cells, center) and large structures (> 20 cells, right) was determined from cultures of patient cohorts (filled circle) and controls (filled tringle) based on n = 50–200 structures per experiment. Repeat experiments for patients were averaged with each value representing one individual (or pool, controls only). Median values of cohorts are indicated. **B** Representative spheroids (two each) for controls and patient cohorts stained for nuclei (blue), apical actin (red), E-cadherin (green) and acetylated tubulin (not shown). Note that projection of central planes, in contrast to 3D stacks as available for evaluation of each structure, may not allow a fair assessment of narrow lumina. Size bar 20 µm. **C** Quality of lumen formation and incidence of non-polarized aggregates in patient cohorts. Assessment of epithelial spheroids was based on 3D structures (> 8 cells) allowing identification of apical surface and (initiation of) lumen, as exemplified in left panel using same 3D structures as in (**A**) [23]. Incidence of defective 3D structures (aggregates) without established epithelial polarity and lumen reveals defective epithelial sheet formation (morphogenesis) of single, primary epithelial cells. Filled circles represent URECs of patients with hereditary cystic kidney diseases, based on 2–3 repeat experiments for 5 of the ARPKD and 1 of the NPH patients. Note, high tendency for defective epithelial morphogenesis in UREC 3D-culture of ARPKD as compared to NPH and BBS cohorts

Spheroids grown out of samples from NPH patients usually met the expected dimension of 8–20 cells, while only a minority of cells led to smaller or larger structures, Fig. 3A. In UREC samples from ARPKD patients, the fraction of spheroids containing > 20 cells was remarkably higher, suggesting higher proliferative capacity. In contrast, UREC spheroids of BBS patients rather tended to be small (< 8 cells), suggesting comparably low proliferation in 3D culture and failure at the level of spheroid establishment, Fig. 3A. Results in the control cohort were inconclusive mainly owing to a restricted quantity and quality of yielded preparations and thus did not allow for any conclusions. An overview of representative 3D structures with lumen for all 4 cohorts is provided in Fig. 3B.

To evaluate the quality of epithelial morphogenesis, the proportion of spheroids (> 8 cells) containing a proper lumen and polarity was assessed for each cohort. This revealed substantial differences between the individual groups with a high incidence of defective UREC structures derived from ARPKD patients, while cells of both—NPH and BBS patients—generated 80–90% intact polarized epithelial structures, Fig. 3C. Owing to the limited number of accessible culture samples and related genotypes as well as against the polygenic background of the analyzed disease spectrum, correlation of 3D epithelial cell properties with the severity of the underlying genetic variants was not possible for BBS and NPH. However, in ARPKD derived samples, loss-of-function variants of *PKHD1* appeared to directly affect the morphogenesis capacities of URECs and thus might provide a promising readout for epithelial function of individual gene variants.

## Discussion

We explored the cellular potential of UREC preparations using pediatric patient cohorts of hereditary cystic kidney diseases manifesting in childhood (ARPKD, NPH and BBS) that mirror representative genetics and typical clinical parameters. The UREC protocol allowed preparation of cells with reproducible yield and quality from pediatric patients, in particular with ARPKD and NPH genetics. Function of these primary cells was addressed in epithelial monolayers using 2D and 3D culture, revealing distinct characteristics related to genetics of the disorder. Defective epithelial morphogenesis, as observed in URECs of ARPKD patients, provides an example of an epithelial (cell) property suitable for testing of pharmaceutical intervention. URECs with BBS genetics showed reduced proliferation in 3D culture with low spheroid numbers suggestive of defective epithelial regeneration. Based on our experience with a range of individual gene variants from different rare diseases, we propose that use of primary UREC culture addressing behavior and defective function of epithelial monolayers, can become a relevant tool for drug testing and personalized medicine.

### What could be the advantages of UREC-based cell biological assays?

Function of human epithelia with cystic kidney disease genetics can also be addressed in organoid culture of differentiated progenitor cells. These allow mechanistic analysis of cyst formation and provide conditions potentially closer to the in vivo situation [24–26]. The approach, however, applies and depends on protocols for cell differentiation, and operates on a time scale of weeks to months making it rather arduous to study disease-causing gene defects in the context of patient’s genotype.

### Time consumption and availability favor use of primary cells

The UREC protocol requires remaining kidney function of patients and provides preparations of differentiated tubular epithelial cells within two weeks of sample collection. After testing for basic characteristics, quality of cell–cell junctions and proliferation potential, URECs can be employed in 3D culture experiments to study formation of monolayered, polarized epithelial spheroids requiring 4–6 days. The assay (1) provides information on cell proliferation in a microenvironment mimicking in vivo conditions (ECM/stiffness/growth factors etc.) and (2) tests the capacity of epithelial cells carrying individual pathogenic variants to perform epithelial morphogenesis and generate correctly polarized epithelia [15, 27]. With appropriate controls in place, this setup will (3) furthermore allow stimulation of cyst formation as currently used in cell-based assays that employ established tubular epithelial cell lines, such as pl-MDCK and mIMCD-3, and thus can provide access to pharmacological testing [16, 18].

### Reproducibility and yield of UREC preparations are variable and may depend on the state of renal epithelia

Establishment of primary cell culture requires a strong expansion of cells for a period of 12–14 days in culture conditions. Our failure to establish UREC culture from urine of healthy children on a regular basis, is most likely due to a minimal requirement of about 1000–10,000 founder cells needed to start a culture. Based on yields observed in this study, a crude estimate would suggest 10–100 times more epithelial cells on the average in urine samples of pediatric patients with hereditary cystic kidney diseases as compared to age-matched healthy controls. This observation most likely reflects enhanced release of tubular renal epithelial cells from diseased or otherwise stressed tissue.

### Is there a dominant effect of pathogenic variants on cell proliferation?

Development of cystic kidney disease can be associated with a massive expansion of kidney epithelia typically over longer periods of time, and although not unchallenged, some studies suggest a proliferative advantage of epithelial cells with hereditary cystic kidney disease-related gene defects [28–30]. In 2D culture and low serum conditions, UREC preparations did not provide evidence for a strong proliferative advantage of specific variants or patient cohorts, and thus can also not account for differing cell yields as observed for patients and controls.

### Which type of kidney epithelial cells is collected and amplified?

Urine contains a variable and wide range of different cell types and urinary single-cell profiling, in a recent study, demonstrated that all types of kidney cells can be detected in urine samples [31]. Establishment of UREC cultures, as described in Methods, relies on selective growth conditions that allow survival of tubular kidney epithelial cells growing in small colonies [11, 22]. This protocol is not selective for specialized epithelial cells within different segments of the nephron, and thus, is expected to allow culture of a mixture of differentiated tubular kidney epithelial cells.

When using cells from ARPKD patients in our laboratory to establish experimental procedures, detection of aquaporin 2 (AQP2)—a water channel and marker of collecting duct epithelia—was to be expected owing to the typical collecting duct defect in respective patients. These defects probably facilitate exfoliation of epithelial cells into urine. However, preparations of NPH and BBS cohorts as well as controls also stained positive for APQ 2. This could be explained by either a bias of the protocol and culture media favoring collecting duct epithelial cells, or by preferential release of cells from that region.

In prolonged culture, media conditions can alter differentiation status of cells, which may explain detection of aquaporin 1 in some cultures in addition to AQP 2. In contrast, expression of megalin, a marker of proximal tube origin [10, 11, 32], is readily lost in culture of primary cells and was not regularly detected. Although in this study, cell culture mainly displayed features of collecting duct epithelial cells, URECs should neither be expected to reliably report epithelial features of a specific nephron segment nor to reveal the acute disease state of epithelia, but rather reflect general features of tubular kidney epithelium.

### Repeated preparations display stable and reproducible cell characteristics providing defined readout for experiments

UREC preparations consist of primary cells which regularly die after 4 weeks, and in our hands, show uncompromised behavior until 21–23 days in culture, allowing cell-based experiments for 7–10 days. This property, while limiting use and availability of cells, also defines viability and reproducible differentiation states. As observed for repeated preparations from several ARPKD patients in the spheroid assay, URECs maintained their characteristics independent of the time point of urine collection during the natural course of disease i.e. early or late stage of ARPKD. While the observation is clearly limited by the comparably low number of repeat preparations, it still suggests that defects of epithelia cell function are linked to patient genetics as required for ex vivo testing.

### How can assay conditions be controlled and standardized?

Experimental setup for spheroid formation in 3D culture was initially derived from experience with MDCKII, canine tubular renal epithelial cells, and further protocols [23, 33, 34]. Establishment of assay conditions and control of experiments with reference cells were made possible through repeated UREC preparations from individuals such as pediatric ARPKD patients with slow disease progression that could provide multiple urine samples over a period of years with consistent yield and quality of cells. Furthermore, storage of UREC preparations at liquid nitrogen temperature without apparent loss of properties allowed timing of and comparison between experiments.

### Experimental controls require establishment of UREC-derived cell lines

All attempts to generate primary cells from healthy pediatric controls in a reproducible, defined fashion did not lead to satisfactory outcome thereby stimulating search for alternatives. In a collaboration with Bert van den Heuvel (Univ. of Leuven) and colleagues, we tested transformation of URECs using a temperature-sensitive mutant of SV40 large T and the essential catalytic subunit of human telomerase hTERT [35]. The approach allowed selection of conditionally immortalized UREC clones from healthy donors and their amplification at 33 °C. Upon induction of differentiation at 37 °C, UREC clones re-acquired expected epithelial morphology, however, concomitantly lost their capacity to proliferate, thus precluding use of these UREC clones in epithelial 3D culture (unpublished observation).

While generation of stably transformed UREC lines can provide access to cell numbers as needed for biochemical and cell biological analysis, a key concern is that signaling events controlling cell adhesion and metabolism, both of which were proposed to contribute to defective kidney epithelia, become altered alongside [30, 36–38]. Therefore, methods to generate UREC cell lines should be standardized and allow close control of alterations. We have promising initial results with UREC clones (iUREC) generated by lentiviral transduction protocols that aim to maintain differentiation state of cells, while alleviating limits in number of cell divisions. The technique is being applied successfully to a growing number of different mammalian and human tissues [39].

## Conclusions

Cultivation of URECs provides a unique tool to analyze epithelial cell function of specific gene variants causing hereditary cystic kidney diseases in the context of the patient’s genotype. Characteristics of cells are reproducible on the level of individual patients and convey cohort similarities. They allow access to (1) the genetic control of disease genes e.g. mRNA levels, splicing etc., to (2) the epithelial defect, and prospectively to (3) the response of patient cells to pharmaceutical intervention. Future research should be directed to generate UREC reference cell lines of ARPKD, NPH, and BBS patients, and also healthy controls allowing assay development in disease modeling and compound testing. Testing of primary URECs in established assays, which is accompanied by suitable reference cell lines, can become a prospect for personalized treatment of pediatric patients suffering from hereditary cystic kidney diseases.

## Methods

### Cell culture media

*Primary medium* (day 1–3), mixture (1 + 1) of DMEM high Gluc., Glucose 4,5 g/l (#41965–039; Gibco, Darmstadt, Germany), HAM’s F12 nutrient Mix (#N6658; Sigma-Aldrich, Taufkirchen, Germany), 10% f.c. fetal bovine serum (FBS) (Biowest, Nuaillé, France) supplemented with 1 × REGM SingleQuot kit (#CC-4127; Lonza, Köln, Germany), 1.4% f.c. Pen/Strep (#A2213; Biochrom, Berlin, Germany) and 1.4% f.c. Amphotericin B (#A2942; Sigma-Aldrich); *Transport medium*, HEPES-buffered DMEM/HAM’s F12 nutrient Mix F12 (#51445C; Sigma-Aldrich) with supplements as in primary medium; *Proliferation medium* (from day 4) contains Assay medium supplemented with 2% f.c. FBS and Pen/Strep; *Assay medium* REBM, Glucose 1,3 g/l (#CC-3190; Lonza) supplemented with 1 × REGM SingleQuot kit, including 0.5% f.c. FBS (#CC-4127; Lonza).

### Preparation, cultivation and proliferation of URECs

The method of UREC culture follows a protocol designed to generate human induced pluripotent stem cells and was modified to allow preferential growth of tubular renal epithelial cells [11, 22]. To allow remote collection of urine cells, pellets (400 g) were washed twice in phosphate buffered saline (PBS) supplemented with Pen/Strep and Amphotericin B, and resuspended in 2 ml of transport medium. Shipment at ambient temperature does not affect viability of urine cells within 24 h. Steps to establish UREC colonies were as detailed in Zhou et al. 2012 [22], using one well of a collagen-coated (0.1% gelatin solution; gelatine from pork skin, #48722, Sigma-Aldrich) 12-well plate for each preparation. In brief, after initial recovery and stabilization of all viable cells found in urine samples, using a full growth medium for 3 days (primary medium), cultures are shifted to starvation condition (proliferation medium). These conditions allow survival of epithelial cell colonies only, and majority of cellular debris is removed with subsequent gradual, daily medium exchange. After 6–8 days of culture, small colonies of epithelial cells are observed and allowed to continue growth until days 12–16 of culture. By this time, established epithelial cultures are evaluated based on morphology of cells and colonies. Proliferation of URECs in 2D was determined using CellTiter 96® Aqueous One Solution (Promega, Madison, USA) according to manufacturer’s protocol. After seeding, measurement was performed at 6 timepoints from 6 to 60 h in triplicate, doubling time was calculated as 1/slope from linear regression of the ln(blank corrected values). Between days 12–14 of culture, UREC preparations can be frozen in Assay Medium (with 45% f.c. FBS, 10% f.c. DMSO) at liquid nitrogen temperature for later use.

A potential limitation to the collection of UREC cells from patients with hereditary cystic kidney disease is recurrent urinary tract infection, leading to contamination of urine samples, which cannot be controlled by standard antifungal and antibiotic treatment of cell culture. In our experience, infections constituted a major obstacle to the collection of UREC cells from adult ADPKD patients but did not pose a problem in our pediatric patient cohorts. Occasional contamination was frequently susceptible to Ciprofloxacin (10 µm/ml f.c.) treatment for 1 week.

### UREC epithelia in 2D and 3D culture

#### 2D culture

Dense monolayers were grown in 12-well chamber slides (removable) (#81201, Ibidi, Martinsried, Germany), fixed for 20 min in 4% paraformaldehyde in PBS, permeabilized for 15 min in 0.25% Triton-X-100 in phosphate-buffered saline (PBS), blocked with 5% normal goat serum (Merck Millipore, Darmstadt, Germany) in PBS and stained cell–cell junctions, polarity or markers of origin, F-actin and nuclei, and mounted with coverslips using Shandon Immu-Mount (Thermo Fisher Scientific, Waltam, MA, USA). Antisera were rabbit anti-E-cadherin (#3195, 24E10; Cell Signaling, Frankfurt, Germany), mouse anti-zonula occludens-1 (#339100, ZO1-1A12; Thermo Fisher Scientific), rabbit anti-pericentrin (#ab4448; Abcam, Cambridge, United Kingdom), mouse anti-acetylated tubulin (6-11B-1; Sigma-Aldrich), mouse anti-aquaporin 1(#NB600-749, 1/A5F6; Novus Bio, Abingdon, United Kingdom), rabbit anti-aquaporin 2 (TA335241; OriGene, Herford, Germany), Alexa Fluor 488 donkey anti-mouse lgG (H + L) (A-21202), Alexa Fluor 555 goat anti-mouse lgG (H + L) (A-21422) and Alexa Fluor 555 goat anti-rabbit lgG (H + L) (A-21428; Life Technologies, Carlsbad, CA, USA). Alexa Fluor 660 Phalloidin, (A-22285; Life Technologies) was used for F-actin and DAPI (0.25 µg/ml; Sigma-Aldrich) to stain nuclei.

#### 3D culture

Culture of URECs in matrigel was adapted from Giles et al. [34]. In brief, 10,000 cells suspended in proliferation medium (100 µl) were mix with equal amount of growth factor reduced, phenolred free matrigel (#356231; Corning, Kaiserslautern, Germany) and filled into 8-well µslides (#80827, Ibidi, Martinsried, Germany). After brief spin (90 g), matrigel was allowed to solidify (15–30 min), overlayed with 200 µl proliferation medium and cultures kept in standard conditions, 5% CO_2_. Assay medium was used for 48 h before fixation. After 6 days, matrigel was extracted and 3D structures fixed for 30 min, using fresh 4% paraformaldehyde in PBS. Step was repeated if necessary. 3D structures were washed in PBS, permeabilized for 20 min using 0.5% Triton-X-100 in PBS and blocked with 5% normal goat serum in PBS. Cells were labelled for cell–cell junction and markers of polarity, and stabilized with Ibidi mounting medium (Ibidi). When kept at 4 °C in dark chamber, stained structures are stable for several months in open wells. For antiserea and fluorescent markers used, refer to 2D culture.

### Imaging 2D/3D cultures and classification of 3D structures

Images were acquired on the Zeiss Axio Observer Z1 microscope, using the 63 × Plan-Apochromat (NA 1.4) oil, the 40 × LD Plan-Neofluar (NA 0.6) and the 20 × Plan-Apochromat (NA 0.8) objectives, the AxioCam MRm Rev.3 camera, and the software package AxioVision version 4.8.2 (all from Zeiss, Göttingen, Germany). Filter sets and related staining were as follows: (1) Alexa Fluor 488—filter set 38 HE, (2) Alexa Fluor 555—filter set 43 HE, (3) Phalloidin-Alexa Fluor 660—filter set 50, and (4) DAPI—filter set 49 (all filter sets from Zeiss). Summary images of spheroids were acquired by fluorescence microscopy in four colors with 35 z-slices per spheroid. *Classification of spheroids*: Spheroid 3D structure, polarity and lumen formation was determined manually on blinded/masked images by three raters using 4-color z-stacks, as described previously [23], and discrepant rating discussed, before unmasking and calculation of distribution.

### Statistical analysis

To compare data set of patient cohorts, multi comparison analysis was performed, if three or more independent patient probes were available in all groups. Dependent on outcome of normality test (Shapiro–Wilk), data sets were analyzed using 1-way analysis of variance (ANOVA) followed by Tukey’s multiple comparison (*p* < 0.05), or Kruskal–Wallis followed by Dunn’s multiple comparison (*p* < 0.05) for non-parametric analysis. All tests were performed using GraphPad Prism version 9 for Windows or MacOS.

## Supplementary Information

**Additional file 1: Fig. S1.** Distribution of kidney volumes within the different patient groups. Filled circles give values of right kidney (A) and total kidney (both organs) (B) as body surface-area related kidney volumes (RV/BSA) [21]. Median values of cohorts and normal range of body-surface-area related renal volume for one organ (grey bar) are indicated. Statistics for (A) *p* < 0.05, Kruskal-Wallis, Dunn’s and (B) *p* < 0.05, ANOVA, Tukey’s confirm increased kidney size for both organs in ARPKD as compared to NPH and BBS cohorts. Distribution of values corresponds to expected range for respective patient cohorts. **Table S1.** Distribution of gene variants.

## Data Availability

The dataset used and/or analyzed during this current study are available from the corresponding author on reasonable request.

## Abbreviations

2D/3D culture: 2/3 Dimensional culture of cells
ADPKD: Autosomal dominant polycystic kidney disease
ANOVA: Analysis of variance
AQP2: Aquaporin 2
ARPKD: Autosomal recessive polycystic kidney disease
BBS: Bardet Biedl syndrome
ECM: Extracellular matrix
eGFR: Estimated glomerular filtration rate
ESKD: End-stage kidney disease
f.c.: Final concentration
FBS: Fetal bovine serum
hTERT: Essential catalytic subunit of human telomerase
mIMCD-3: Mouse inner medullary collection duct 3
NEOCYST: German network for early onset cystic kidney disease
NPH: Nephronophthisis
*NPHP1*: Nephrocystin1
PBS: Phosphate-buffered saline
*PKHD1*: Polycystic kidney and hepatic disease gene 1
pl-MDCK: Principal-like Madin-Darby canine kidney
URECs: Urine-derived renal tubular epithelial cells
Zo-1: Zonula occludens 1
