# Supplementary material for: Primary URECs: a source to better understand the pathology of renal tubular epithelia in pediatric hereditary cystic kidney diseases
Source: Orphanet J Rare Dis. 2022 Mar 9;17:122. doi: 10.1186/s13023-022-02265-1 (PMC8905910; doi:10.1186/s13023-022-02265-1)
Supplement: Supplementary file 1 — Additional file 1: Fig. S1. Distribution of kidney volumes within the different patient groups. Filled circles give values of right kidney (A) and total kidney (both organs) (B) as body surface-area related kidney volumes (RV/BSA) [21]. Median values of cohorts and normal range of body-surface-area related renal volume for one organ (grey bar) are indicated. Statistics for (A) p < 0.05, Kruskal-Wallis, Dunn’s and (B) p < 0.05, ANOVA, Tukey’s confirm increased kidney size for both organs in ARPKD as compared to NPH and BBS cohorts. Distribution of values corresponds to expected range for respective patient cohorts. Table S1. Distribution of gene variants. [file 13023_2022_2265_MOESM1_ESM.pdf]

Additional file 1

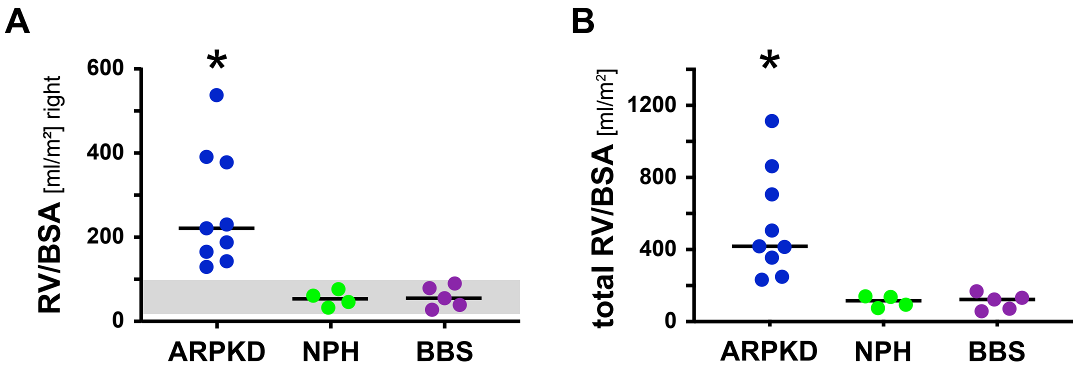

Fig. S1 Distribution of kidney volumes within the different patient groups. Filled circles give values of right kidney (**A**) and total kidney (both organs) (**B**) as body surface-area related kidney volumes (RV/BSA) [21]. Median values of cohorts and normal range of body-surface-area related renal volume for one organ (grey bar) are indicated. Statistics for (**A**) $p<0.05$, Kruskal-Wallis, Dunn's and (**B**) $p<0.05$, ANOVA, Tukey's confirm increased kidney size for both organs in ARPKD as compared to NPH and BBS cohorts. Distribution of values corresponds to expected range for respective patient cohorts.

Distribution of gene variants

patient	phenotype	gene variant(s)	eGFR [mL/min/1.73m^2]
ARPKD 1	ARPKD	compound heterozygote PKHD1; missense (p), splice defect (m)	35
ARPKD 2	ARPKD	compound heterozygote PKHD1; missense (p, m)	45
ARPKD 3	ARPKD	compound heterozygote PKHD1; missense (p, m)	40
ARPKD 4	ARPKD	compound heterozygote PKHD1; nonsense (p), in-frame deletion, (m)	32
ARPKD 5	ARPKD	compound heterozygote PKHD1; missense (p, m)	30
ARPKD 6	ARPKD	compound heterozygote PKHD1; missense (p, m)	137
ARPKD 7	ARPKD	compound heterozygote PKHD1; missense (p), nonsense (m)	119
ARPKD 8	ARPKD	not available	45
ARPKD 9	ARPKD	compound heterozygote PKHD1; nonsense, missense (no segregation)	40
ARPKD 10	ARPKD	not available	35
NPH 1	Nephronophthisis	homozygote NPHP1, deletion exons 1-20	16
NPH 2	Nephronophthisis	homozygote NPHP1, deletion	13
NPH 3	Nephronophthisis	homozygote NPHP1, deletion	21
NPH 4	Nephronophthisis	compound heterozygote NPHP3, missense (p), nonsense (m)	5

Table S1-1

Distribution of gene variants (continued)

patient	phenotype	gene variant(s)	eGFR [mL/min/1.73m^2]
BBS 1	Bardet Biedl Syndrome	homozygote BBS7, missense	46
BBS 2	Bardet Biedl Syndrome	homozygote BBS7, missense	69
BBS 3	Bardet Biedl Syndrome	homozygote BBS10, out-of-frame deletion, STOP	64
BBS 4	Bardet Biedl Syndrome	homozygote BBS1, missense	103
BBS 5	Bardet Biedl Syndrome	homozygote BBS4, deletion exons 7+8	86

Table S1-2
